# The Development of a Chocolate-Based Chewable Tablet of Prednisolone—Enhancing the Palatability of Steroids for Pediatric Use

**DOI:** 10.3390/pharmaceutics16081099

**Published:** 2024-08-21

**Authors:** Okhee Yoo, Edith Tang, Md Lokman Hossain, Britta S. von Ungern-Sternberg, David Sommerfield, Chloe Heath, Neil Hauser, R. Nazim Khan, Cornelia Locher, Minh Nguyen, Lee Yong Lim

**Affiliations:** 1 Discipline of Pharmacy, School of Allied Health, The University of Western Australia, Perth, WA 6009, Australia; okhee.yoo@uwa.edu.au (O.Y.); edith.tang@uwa.edu.au (E.T.); mdlokman.hossain@uwa.edu.au (M.L.H.); connie.locher@uwa.edu.au (C.L.); minh.nguyen@uwa.edu.au (M.N.); 2 Institute for Pediatric Perioperative Excellence, The University of Western Australia, Perth, WA 6009, Australia; britta.regli-vonungern@health.wa.gov.au (B.S.v.U.-S.); david.sommerfield@health.wa.gov.au (D.S.); neil.hauser@health.wa.gov.au (N.H.); nazim.khan@uwa.edu.au (R.N.K.); 3 Perioperative Medicine Team, Perioperative Care Program, Telethon Kids Institute, Perth, WA 6009, Australia; 4 Division of Emergency Medicine, Anaesthesia and Pain Medicine, Medical School, The University of Western Australia, Perth, WA 6009, Australia; 5 Department of Anaesthesia and Pain Medicine, Perth Children’s Hospital, Perth, WA 6009, Australia; 6 Department of Mathematics and Statistics, The University of Western Australia, Perth, WA 6009, Australia

**Keywords:** medication adherence, paediatric formulation, taste masking, prednisolone, chocolate-based delivery system

## Abstract

Oral liquid prednisolone medications have poor acceptance among paediatric patients due to ineffective masking of the bitterness taste of prednisolone. This study aimed to develop a child-friendly prednisolone tablet using a patented chewable chocolate-based delivery system (CDS) previously applied successfully to mask the bitterness tastes of midazolam and tramadol. Prednisolone sodium phosphate (PSP) and prednisolone base (PB) CDS tablets were prepared, and the manufacturing process was optimised using a design of experiments (DoE) approach. Stability was assessed by quantifying residual drug content via a validated HPLC assay. A pilot randomised crossover taste study involving 25 young adult volunteers evaluated taste-masking effectiveness against Redipred™, a commercial oral PSP liquid medicine. The results showed that the PSP CDS tablet was chemically stable following storage for three months at ambient temperature, while the PB CDS tablet was unstable. The optimised PSP CDS tablet, manufactured at 50 °C with a stirring time of 26 h, was found to release over 80% of its drug load within 20 min in 0.1 M HCl and had a significantly better mean taste score compared to Redipred™ (7.08 ± 2.40 vs. 5.60 ± 2.33, *p* = 0.03). Fifty six percent of the participants preferred the PSP CDS tablet. In conclusion, compared to Redipred™, the CDS technology provided a more effective taste masking of PSP, potentially offering a child-friendly prednisolone formulation with improved compliance, dosing accuracy, and storage stability.

## 1. Introduction

Poor adherence to oral medicines is an ongoing problem in the paediatric population and it is often due to a lack of child-friendly taste masked formulations. A drug that is commonly prescribed for children but presents problems with therapy adherence due to its aversive taste is prednisolone. Prednisolone is a synthetic corticosteroid prescribed for children at a daily dose of 1–2 mg/kg to manage a range of autoimmune and inflammatory diseases. It is commonly administered in the perioperative setting to children with unstable asthma, bronchial hyper-reactivity, chronic inflammation, or autoimmune diseases. Oral prednisolone at a dose of 1 mg/kg/day for 3 days also reduced the length of hospital stays for children aged 2–6 years presenting with virus-associated wheeze, with the greatest efficacy seen in children with severe wheeze and a prior history of asthma [1].

**Figure 1:**
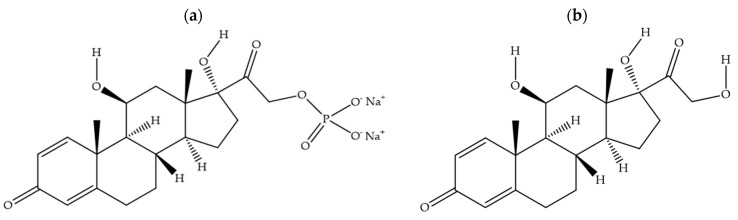
Chemical structures for prednisolone sodium phosphate (**a**) and prednisolone base (**b**).

Prednisolone medicines for oral administration are available as tablets (1 mg, 5 mg, or 25 mg prednisolone per tablet) and liquids (5 mg prednisolone in each mL) and are formulated using either prednisolone sodium phosphate (Figure 1a) or prednisolone base (Figure 1b). Parents prefer the liquid formulations for young children, even though studies have consistently shown oral prednisolone liquids to be poorly tolerated due to their unpalatable taste [2,3,4,5]. The oral prednisolone liquid medicines also have a short shelf life of 4 weeks after the bottle is opened. As an alternative, carers could crush the prednisolone tablets into a powder form for administration to children unable to safely swallow whole tablets. This practise, however, has the potential to increase dosing error, and it also exposes the bitterness taste of prednisolone, which is not effectively masked with co-administration of food like applesauce, chocolate syrup, or custard. In one study, 23% of child participants had to be withdrawn from treatment due to repeated vomiting associated with the consumption of crushed prednisolone tablets mixed with food [6]. As poorly tolerated medicines have the potential to lead to therapeutic failure, age-appropriate formulations of prednisolone are urgently needed.

Our team has developed a chewable chocolate-based delivery system (CDS) that has been proven through two paediatric clinical trials to effectively mask the bitterness taste of midazolam and tramadol without compromising the bioavailability of the drugs [7,8]. The CDS platform primarily uses chocolate as a main ingredient, which serves as an effective taste-masking agent by enveloping the active ingredient in a palatable matrix [9]. Its high cocoa content, smooth texture, and familiarity as a food ingredient help to make the final formulation more acceptable in taste, particularly for paediatric patients. The taste-masking efficiency is further enhanced by combining the chocolate with other taste-enhancing ingredients such as sodium chloride, xanthan gum, and Cremophor RH40 in the final CDS formulation [9]. The collective data suggest that the chewable CDS tablets offer several other advantageous features, including improved dosing accuracy, storage stability at ambient temperature, and the option to convert the chewable tablets into a palatable liquid form by simply adding warm water [10]. Based on these promising findings, the CDS platform might also provide a potential formulation to prepare chewable taste-masked prednisolone tablets.

The aim of this study was to evaluate the transferability of the CDS platform to prednisolone and, for the first time, directly compare the taste masking effectiveness of the CDS platform with a commercial paediatric oral liquid in a randomised, cross-over taste study. CDS tablets containing 10 mg of prednisolone were prepared using prednisolone base and its ester derivative prednisolone sodium phosphate, as both drug molecules are used to prepare the commercial prednisolone medicinal products. The CDS platform is scored and can be halved, allowing the prednisolone 10 mg CDS tablet to offer 5 mg dose increments. This feature enables precise dose adjustments for target patients aged 3 years and older to administer a dosage of 1–2 mg/kg. The CDS platform has a chocolate base, and as the process of conching (shearing and aeration of chocolate mass at temperatures above 40 °C [11]) influences the physicochemical and sensory characteristics of chocolate products [12], the stirring temperature and stirring time in the manufacturing process for the CDS tablets was optimised using the Design of Experiments (DoE) approach. Additionally, a pilot randomised open-label cross-over taste study involving young adult healthy volunteers was conducted to evaluate the taste masking effectiveness of the optimised prednisolone CDS tablet against Redipred™, a commercial oral prednisolone liquid.

## 2. Materials and Methods

### 2.1. Materials

Prednisolone sodium phosphate (PSP) and prednisolone base (PB), both of USP grade, were obtained from Medisca (Mascot, NSW, Australia). Excipients in the CDS tablets were polyethylene glycol (PCCA, Matraville, NSW, Australia), hydrogenated polyoxyl 40 castor oil (Cremophor^®^ RH40, BP grade, Ingredient Plus, Rydalmere, NSW, Australia), xanthan gum (PharmAust, Malaga, WA, Australia), steviol (100% pure organic stevia extract powder, Hemmant, QLD, Australia), and Jamaica dark chocolate (Springer Foods, Myaree, WA, Australia). Chemicals used in the study include methanol (Macron Fine Chemicals, Center Valley, PA, USA), hydrochloric acid (Scharlau, Barcelona, Spain), hydrogen peroxide (Ajax Finechem, Sydney, NSW, Australia), acetonitrile, and sodium hydroxide pellets (AR grade, Merck, Darmstadt, Germany). Double deionised water (PSI Water Filters, South Launceston, TAS, Australia) and HPLC-grade organic solvents were used throughout.

### 2.2. Methods

#### 2.2.1. CDS Tablet Preparation and Storage Stability Study

Formulations for the CDS prednisolone tablets were adapted from the tramadol CDS formulation [10]. The tramadol HCl content (11.3 mg) was replaced with 13.44 mg of prednisolone sodium phosphate to prepare the PSP CDS tablet, and with 10 mg of prednisolone base to prepare the PB CDS tablet. For each CDS tablet, the chocolate mass was adjusted accordingly to prepare the final formulations (Table 1). We tested five different types of chocolate bases to formulate the prednisolone CDS tablets, ultimately selecting the Jamaica dark chocolate based on a preliminary taste evaluation conducted by our formulation team. The Jamaica dark chocolate had comparable components to the other chocolates, and its cocoa butter content was 20% compared to the range of 15–58% for the other four chocolates while its total fat content of 30% was similar to the other chocolates tested. The final ingredients are specified in Table 1, with the PSP and PB contents both delivering the equivalent of 10 mg of prednisolone per CDS tablet. A placebo CDS tablet void of the active drug was also prepared (Table 1). For each formulation, the ingredients were mixed by stirring for 16 h in a beaker maintained at 55 °C on a magnetic hot plate (Radleys, Essex, UK), following which the molten liquid was poured into calibrated plastic moulds (R.D. troche 0.5 mL mould, PCCA, Matraville, NSW, Australia) and allowed to solidify at room temperature. The solidified tablets were demoulded, individually wrapped in foil, and stored in plastic containers at ambient conditions and 40 °C (UF160 Oven, Memmert, Schwabach, Germany). The drug content in the CDS tablets was monitored using a validated HPLC assay at baseline and at 1 month and 3 months of storage to determine the chemical stability. The tablets were deemed stable if the residual drug content did not fall below 90% of the baseline drug content before storage.

#### 2.2.2. Development and Validation of Drug Assay

The two drugs were quantified using the high-performance liquid chromatographic (HPLC) method described in the British Pharmacopoeia, with modifications. Analyses were performed on an Agilent 1260 Infinity instrument (Agilent Technologies Australia, Chatswood, NSW, Australia) equipped with a diode-array detector and Xbridge C18 column (5 μm, 150 mm, 4.6 mm, Waters Australia, Rydalmere, NSW, Australia). Gradient elution was employed using a mobile phase consisting of acetonitrile and pH 7.4 phosphate-buffer solution, as described in Table 2. The flow rate was 0.8 mL/min and 10 µL of the samples was injected for analysis for a total run time of 35 min. The drugs were detected at a wavelength of 254 nm. All samples were filtered (0.45 μm nylon filter, Agilent, Lexington, MA, USA) prior to analysis. Chromatographic data were analysed using Agilent ChemStation software (OpenLAB C01.07, version 02.05.021, Knauer, Berlin, Germany).

The HPLC method was validated according to ICH guidelines [13]. Linearity was established (r^2^ > 0.999) for the calibration standards of PSP (6.8–34 ng/mL) and PB (4.8–24 ng/mL). Calibration standards were prepared from stock solutions (0.34 mg/mL PSP; 0.24 mg/mL PB) using 60% methanol in deionised water (60% MeOH) as a solvent. Accuracy and precision were assessed by analysing in triplicate the 50%, 80%, and 100% concentration points in the calibration range (equivalent to 5, 7.5 and 10 mg of prednisolone) for both PSP and PB. To verify that the assay was stability-indicating, forced degradation studies were conducted with PSP and PB samples subjected to degradation by heating for 1 h at 100 °C in acid (1.0 M HCl) or base (1.0 M NaOH), or by exposure to UV light in 20% (*v*/*v*) hydrogen peroxide for 1 h.

To analyse the drug content in a CDS tablet, the CDS tablet was weighed into a 100 mL volumetric flask, and the tablet was melted by heating at 60 °C with 60 mL of 60% MeOH. The mixture after cooling was then adjusted to volume with 60% MeOH. Accuracy samples were similarly prepared using the placebo CDS tablets and spiking the samples with known amounts (5, 7.5 and 10 mg) of PSP or PB before heating with 60% MeOH. Analysis of the accuracy of the samples also demonstrated assay specificity in the presence of the CDS tablet excipients. The linearity of the calibration curves at higher concentration ranges (68–340 ng/mL for PSP, and 48 to 240 ng/mL for PB) was established to cater for the higher drug loads in these samples.

#### 2.2.3. Optimisation of the Manufacture Process

The CDS tablets were manufactured using a method comparable to conching in chocolate manufacturing [14]. The conching process is aimed at developing the desired flavour and texture of a chocolate product by removing volatiles of undesirable flavours, breaking up particle clumps to minimise grittiness, and evenly coating all particles with molten chocolate and other taste-modifying ingredients. Conching involves slow agitation and careful control of the stirring duration and temperature. The stirring temperature and stirring time were therefore identified to be the critical processing parameters in the manufacture of the prednisolone CDS tablets.

A DoE approach was employed to optimise the manufacturing process, with the stirring temperature and stirring time set up as two numerical factors that were varied independently (Table 3). CDS tablets that effectively masked the taste of tramadol hydrochloride and midazolam hydrochloride were manufactured at a temperature of 55 °C and stirring time of 16 h. These conditions were thus chosen as the central points for the experimental design, and the other points were determined using a systematic variation approach. The lower boundary for the temperature was set at 48 °C, which was the temperature at which all ingredients could be melted within a practical timeframe. The upper boundary for the temperature was therefore set at 62 °C to maintain symmetry around the central temperature value of 55 °C. Similarly, with 16 h chosen as the central point for the stirring time and the lower boundary set at 2 h, which was the minimum stirring time required to mix all ingredients to observable homogeneity, the upper boundary for the stirring time was set at 30 h. A quadratic polynomial model was selected to the response to account for second-order effects. A Factorial/Response Surface Methodology (RSM) was applied, specifically employing a full central composite design consisting of eight non-central and five central points using Stat-Ease 360 (version 23.1.6, Minneapolis, MN, USA).

An appropriate non-linear model was fitted to the setting time against the temperature and stirring time. The tablet setting time was measured at 10 s intervals by using a wooden probe (80 mm length, 2 mm diameter) to determine whether the molten CDS poured into the troche moulds had solidified. The setting time was defined as the time point at which the formulation did not yield when the wooden probe was inserted, and there was no visible residue adhering to the probe on withdrawal from the samples. Nine samples were assessed at each design point. The influence of the stirring time on the tablet’s taste was assessed following the DoE study on setting time. Tablet taste was assessed using a taste panel and is described under ‘Taste Evaluation’.

#### 2.2.4. In Vitro Drug Dissolution Profile of Optimised Prednisolone Sodium Phosphate CDS Tablet

In vitro drug dissolution experiments were performed on the optimised PSP CDS tablet (*n* = 3) at baseline and following storage at ambient condition for 3 months. Dissolution experiments were conducted using an 8-station USP paddle apparatus (Agilent 708-DS Dissolution Apparatus, Lexington, MA, USA). The dissolution medium consisted of 900 mL of 0.1 M HCl maintained at 37 ± 0.5 °C and stirred at 50 rpm. As the PSP CDS tablets were formulated to be chewable tablets (W × L × H = 1 cm × 1 cm × 0.5 cm), tablet mastication was simulated by cutting each CDS tablet into 16 pieces immediately before placing in the dissolution medium. Aliquots of 1 mL were sampled from the dissolution medium at 0, 5, 10, 20, 30, 45, and 60 min for HPLC analysis of drug content. All samples were filtered (0.45 μm nylon filter, Agilent, Lexington, MA, USA) prior to analysis. A sampled aliquot was replaced with an equal volume of fresh dissolution medium at each time point. The cumulative drug release was calculated and plotted against the dissolution time to obtain the drug dissolution profile.

#### 2.2.5. Taste Evaluation

Two separate randomised, open-label human taste evaluations were conducted on the CDS tablets containing 13.44 mg of PSP in each tablet. Study 1 aimed to assess the PSP CDS tablets manufactured with two different stirring times. The taste evaluation assessed a half tablet manufactured at 50 °C with a longer stirring duration of 26 h, and a half tablet with a shorter stirring duration of 6 h. Study 2 compared the taste masking effectiveness of the PSP CDS tablet against a standard of care comparator, Redipred™ (Aspen Australia, St. Leonards, Australia), which is an oral liquid formulation of PSP available in Australia. In this study, a half tablet of PSP CDS (equivalent to 6.72 mg PSB) manufactured under optimal conditions was assessed against 1 mL of Redipred™ (equivalent to 6.72 mg of PSB). Approval for both human taste evaluation studies was obtained from the UWA Human Research Ethics Committee (2023/ET000155).

**Figure 2:**
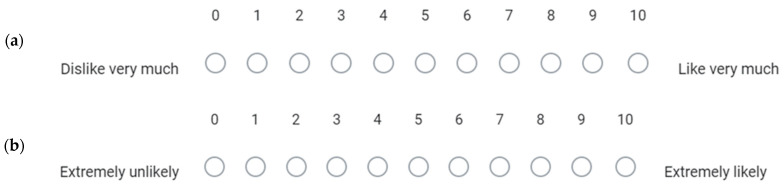
The 11-point rating scales for (**a**) taste evaluation and (**b**) willingness to take again.

Participants in both taste studies were aged 18 to 25 years and had provided written consent. Exclusion criteria included allergy to any ingredients in the CDS tablets or Redipred™ oral liquid, known history of gastrointestinal problems, current illness, currently taking any medication, pregnancy, or breastfeeding. Participants were guided to taste and assess each formulation by employing a ‘chew-and-spit’ method for the CDS tablets and a ‘swill-and-spit’ method for the oral liquid. They were explicitly instructed not to swallow any of the CDS tablet or oral liquid contents. Study 1 and Study 2 were conducted separately, with each study lasting about 20 min for a participant. For each study, the sequence of administering the investigational products was randomised for the participants. Each participant received 600 mL of bottled mineral water and 10 plain crackers to eliminate any residual taste in their mouth before and after evaluating each sample. To minimise the crossover effect of residual taste, participants were instructed to wait 10 min from completing the evaluation of one test sample and commencing on the next test sample. After tasting each sample, participants were asked to rate how much they liked the sample and their willingness to take the formulation again if needed. These assessments were performed using two 11-point number rating scales, as depicted in Figure 2a,b. Additionally, participants were asked to indicate which formulation they would prefer to take again when feeling unwell.

#### 2.2.6. Data Analyses

Drug content and dissolution data were expressed as mean ± SD (*n* = 3). Data obtained in the DoE and taste evaluation studies were analysed in the R statistical environment [15]. Two taste evaluations were conducted: the first compared PSP CDS tablets prepared at two different stirring times (6 h and 26 h), while the second compared the optimised PSP CDS tablet with a commercial PSP oral liquid. For both taste evaluations, a cross-over design was employed where each participant tasted both preparations. Consequently, a linear mixed-effects model with each participant as a random effect was applied to analyse the taste score and score for willingness to take again [16]. The willingness to take again scores were further converted to a binary preference variable and a logistic regression model, applied to determine the preference between the PSP CDS tablet and the commercial PSP oral liquid.

## 3. Results and Discussion

### 3.1. HPLC Method Validation

**Figure 3:**
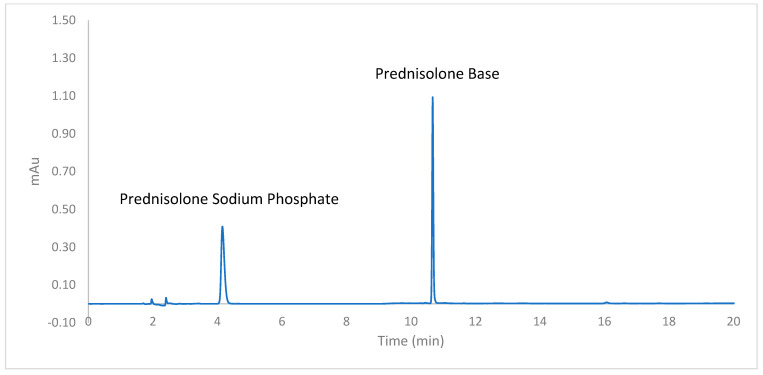
HPLC chromatogram of prednisolone sodium phosphate and prednisolone obtained using the validated HPLC assay.

The HPLC chromatograms showed a sharp and symmetrical peak at the retention time of 4.17 min for PSP and another sharp peak at 10.67 min for PB (Figure 3). The analysis of placebo CDS tablets spiked with the drugs showed a clear resolution of drug peaks from the peaks of excipients used in the CDS formulations, underscoring the specificity of the HPLC assay for PSP and PB. Precision (%RSD < 1.47%) and accuracy (>99% recovery) were well within the 15% limit specified in the ICH guideline for both parameters (Table 4) [13].

**Figure 4:**
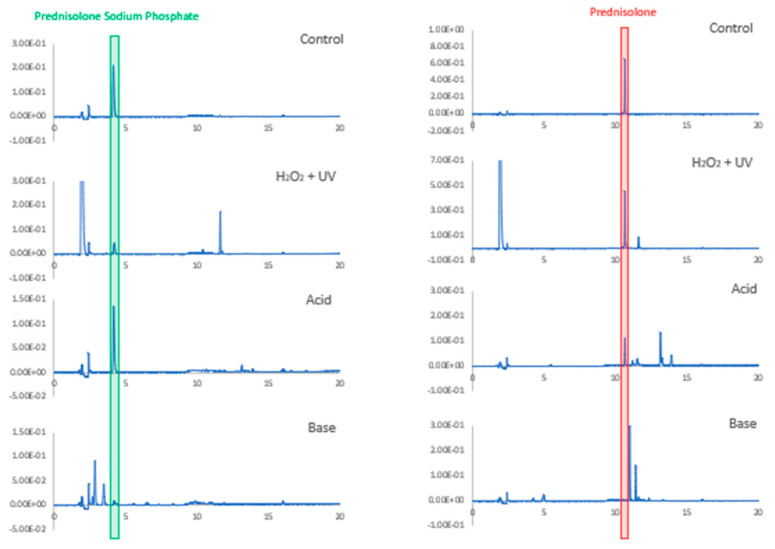
Chromatograms of prednisolone sodium phosphate and prednisolone base before (control) and after being subject to degradations conditions: heating for 1 h at 100 °C in acid (1.0 M HCl) or base (1.0 M NaOH) or exposure for 1 h to UV light in 20% (*v*/*v*) hydrogen peroxide.

Both PSP and PB demonstrated susceptibility to degradation when heated for 1 h at 100 °C with 1.0 M NaOH, while PSP was more resistant than PB to degradation in the corresponding acidic conditions (Figure 4). PB was relatively more stable than PSP when exposed to UV in peroxide. HPLC analyses showed that the drug peaks were well separated from the peaks of the degradation products produced by exposure to acid, base, UV and peroxide, confirming that the assay was stability-indicating (Figure 4).

### 3.2. Drug Stability in the CDS Formulations

PSP CDS tablets and PB CDS tablets were successfully prepared using the formulations (Table 1) and manufacture process described. The baseline drug content exceeded 99% of the labelled content for both tablets (Table 5). The tablets were considered to have acceptable chemical stability if drug content did not fall below 90% of the baseline drug content upon storage. The two CDS tablets fulfilled this requirement at one month of storage at ambient conditions; however, the PB CDS tablets had a residual drug content below 90% of the baseline at three months of storage, indicating chemical instability. The PSP CDS tablet retained 98.6% of the baseline drug content after three months of storage at ambient conditions, demonstrating high chemical stability. At the accelerated stability assessment conditions of 40 °C, the PSP CDS tablet continued to maintain an acceptable residual drug content even after three months, while the instability of the PB CDS tablet was magnified and it retained less than 13% of the baseline drug load at three months.

The CDS matrix contains several ingredients. Based on the literature evidence, the instability of the PB tablet is likely due to the interactions of PB with PEG and Cremophor RH40 present in the CDS matrix. 21-Hydroxy corticosteroids are capable of undergoing degradation in pharmaceutical vehicles containing PEG [17,18,19] or Cremophor RH40 (which contain ethoxyl groups and polyethylene glycol) [18]. Degradation of the corticosteroids results almost exclusively from reactions of the C17-dihydroxyacetone side chain [20], and esterification on the 21-OH moiety may create steric hindrance to confer protection against these degradation pathways. This has previously been demonstrated for prednisolone acetate in vehicles containing Cremophor RH40 [18] and is now demonstrated in the CDS matrix for PSP, which has a phosphate ester group at C21.

The differential chemical stability between the PB CDS tablet and PSP CDS tablet suggests that prednisolone CDS tablets should only be formulated using PSP and not PB. For this reason, further development of the PB CDS tablet was aborted. Optimisation of the manufacture process was conducted on only the PSP CDS formulation.

### 3.3. Design of Experiment

The optimisation of manufacturing was performed only for the PSP CDS formulation and not for the PB CDS tablet, as the PB CDS tablet was found to be unstable upon storage.

**Figure 5:**
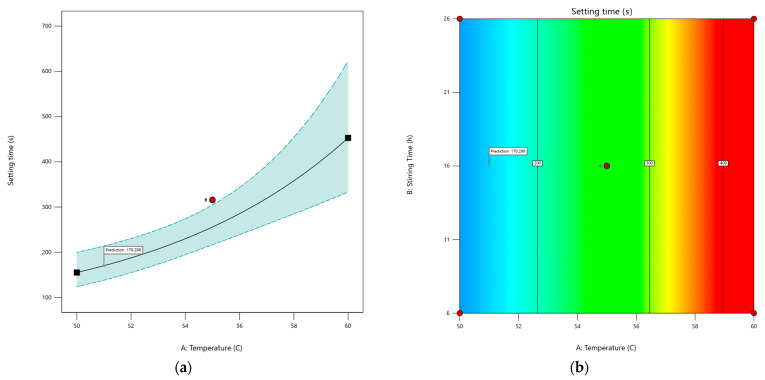
(**a**) An exponential model of predicted setting time as a function of temperature represented by a black solid line, with the dotted line showing the 95% confidence interval. Red dots indicate design points, and the predicted setting time under the optimal temperature is marked with a flag. (**b**) Contour plot of setting time based on temperature and stirring time, showing no effect of stirring time on tablet setting time. Red dots represent design points, the black solid line shows predicted setting time with values in black boxes, and red areas indicate longer setting times compared to blue areas. The predicted setting time under optimal conditions is marked with a flag.

**Figure A1:**
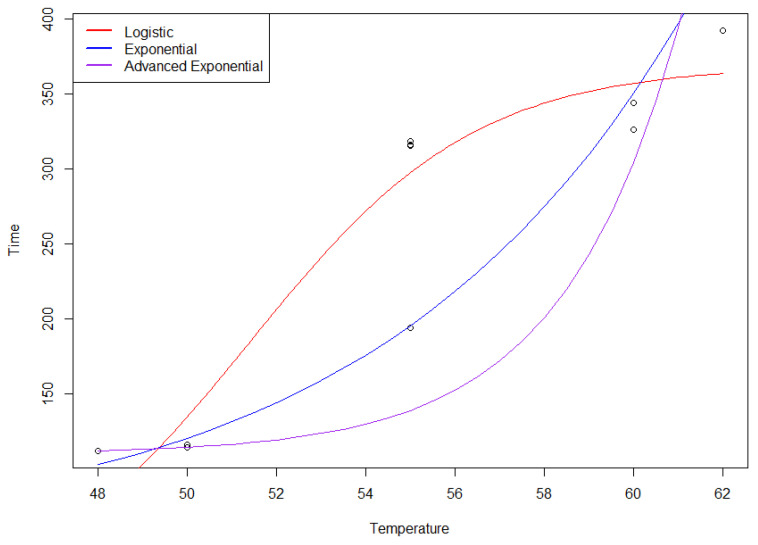
Comparison of logistic, exponential, and advanced exponential models for setting time–temperature relationship.

**Figure A2:**
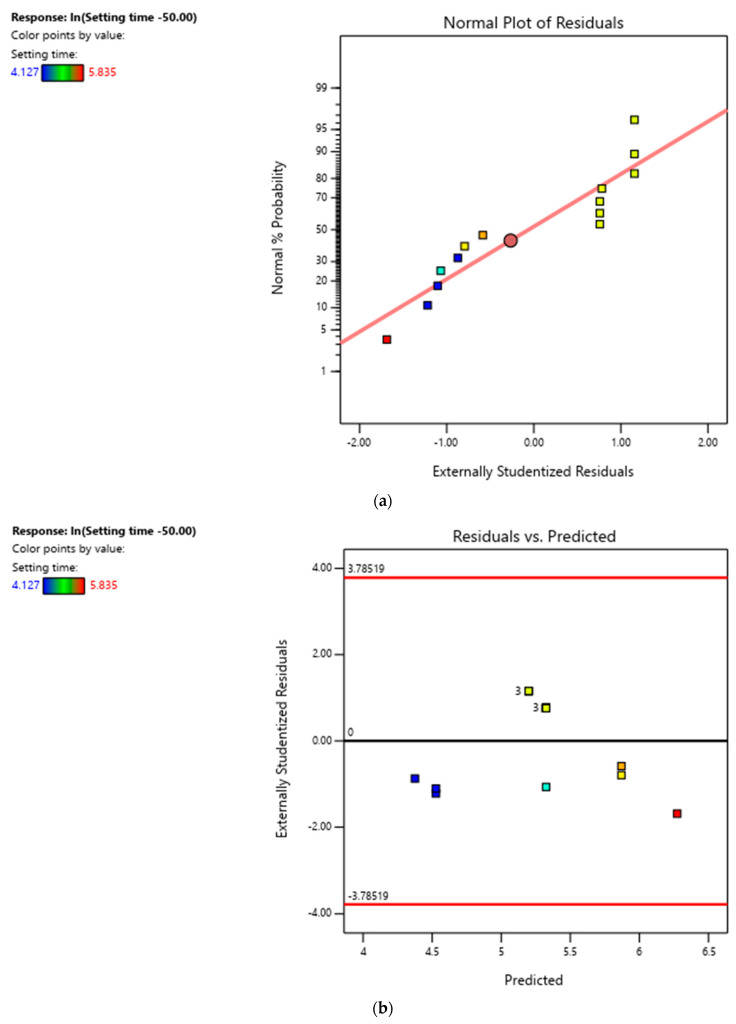

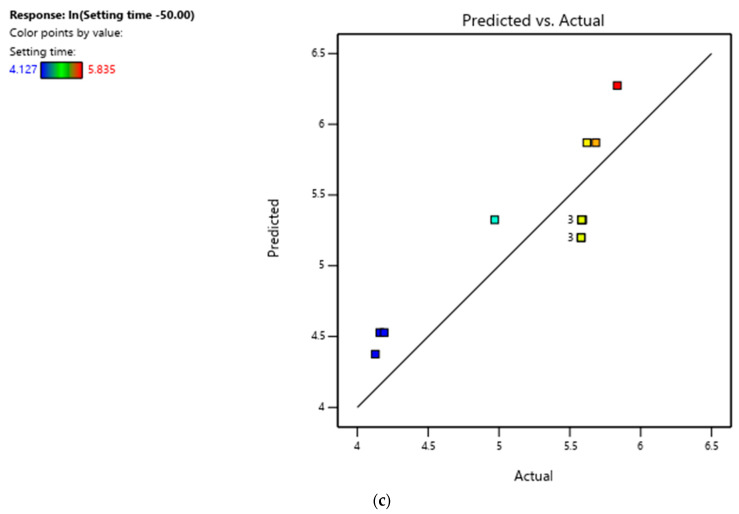
(**a**) Normal plot of residuals for ln(*setting time* − 50.00). (**b**) Residuals vs. predicted for ln(*setting time* − 50.00). (**c**) Predicted vs. actual for ln(*setting time* − 50.00).

The PSP CDS tablet setting times for the different DoE runs are shown in Table 6. The stirring time was found to have no significant effects, with the data plots showing no relationship between stirring time and setting time. The graph of setting time against temperature shows a non-linear plot (Figure 5a). Consequently, a non-linear statistical model was fitted to the data. The shape of the plot indicates a logistic model or an exponential model. The exponential model was deemed appropriate, with a significance level of *p* = 0.00038. The different model plots (Figure A1), along with the development of final model and its diagnostics (Figure A2), are provided in Appendix A. The exponential model plot within the design space indicates that the setting time exhibited a marked increase when the CDS formulation was prepared at elevated temperatures (Figure 5).

From a manufacturing perspective, a setting time that is too brief would cause the molten CDS formulation to solidify before it could be poured into the moulds. Conversely, an excessive setting time would unnecessarily prolong the manufacturing process. For the manufacture of 30 tablets using the troche mould, we determined empirically that a setting time of about 200 s would be needed to prepare the CDS tablets in a 30-cavity troche mould. The target goal was therefore to minimise the setting time to about 200 s. The model predicted a manufacturing temperature of 51 °C with 16 h of stirring will achieve a setting time of 170.29 s.

The effect of stirring time on the taste acceptability of the PSP CDS tablets was determined using tablets manufactured at 50 °C and stirring times of 6 h (abbreviated as T6) and 26 h (T26). The stirring times were set up to be 10 h below (T6) and 10 h above (T26) the optimal stirring time of 16 h, determined previously for the tablet setting time. The taste scores for T6 and T26 were obtained from 28 participants, with a wait time of 10 min between samples. T26 received only a slightly higher mean taste score compared to T6 (mean score ± SD: 5.89 ± 2.20 vs. 5.57 ± 2.18). There were no statistically significant differences between T26 and T6 with regard to the taste scores or the scores for willingness to take the tablet again (*p* = 0.577 and *p* = 0.418, respectively). When asked which formulation they would prefer to take when feeling unwell, 57% of the participants chose T26 rather than T6. Given the slight preference for T26, the final optimised PSP 13.44 mg CDS tablet was prepared at 50 °C with a stirring time of 26 h.

### 3.4. In Vitro Dissolution Profile of Optimised Prednisolone Sodium Phosphate CDS Tablet

**Figure 6:**
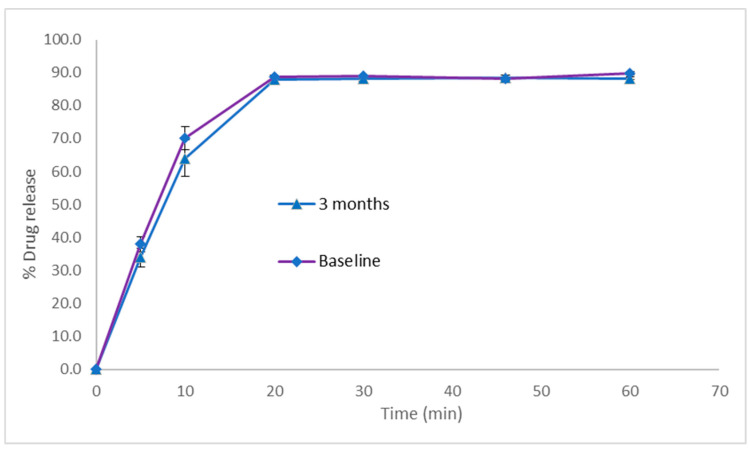
In vitro drug release profiles in simulated gastric fluid for prednisolone sodium phosphate CDS tablets stored at ambient temperature. Dissolution experiments were performed using USP paddle apparatus operating at 50 rpm and 37 °C, with use of 900 mL of 0.1 M HCl as the dissolution medium. Data represents mean ± SD (*n* = 3).

In vitro dissolution experiments revealed that over 80% of the PSP load in the optimised PSP CDS tablets was released at 20 min in 0.1 M HCl, with 90% of the drug load released at 60 min. This dissolution profile did not change for the tablets after 3 months of storage under ambient conditions (Figure 6), indicating a lack of interaction between PSP and the tablet matrix during storage.

PSP is freely soluble in water. According to the FDA guidelines for immediate-release solid oral drug products containing highly soluble drugs, the dissolution criterion stipulates that at least 80% of the drug load should dissolve within 30 min [21]. The optimised PSP CDS tablets therefore successfully met the criteria for an immediate-release dissolution profile, aligning with our development objectives for this formulation.

### 3.5. Taste Evaluation of the Optimised Prednisolone Sodium Phosphate CDS Tablet

The ideal taste panellists for a paediatric formulation would be children; however, recruiting young children to conduct a comparative taste evaluation of multiple prednisolone samples is challenging with regard to obtaining parental consent and achieving consistent adherence to protocol instructions set up to protect participants and increase data reliability. Recruiting healthy young adults between 18 and 40 years of age presents a workable alternative as adults of this age range have been shown to give similar taste scores to children and may be better able to discriminate taste between two products in a cross-over evaluation design [22,23,24].

A total of 25 participants took part in the taste evaluation of the optimised PSP CDS tablet against the commercial oral PSP liquid comparator. The participants tasted half of a PSP CDS tablet and 1 mL of PSP oral liquid, both of which contained an equivalent prednisolone dose of 6.72 mg. The 25 participants were randomised into the CDS–oral liquid group (*n* = 13), which received the CDS tablet followed by oral liquid after a 10 min washout (*n* = 13), and the oral liquid–CDS group (*n* = 12), which received the oral liquid first, followed by the CDS tablet after a 10 min washout.

A linear mixed-effects model was fitted to the taste scores (with each participant as a random factor) and a variance model for the formulation type (CDS or liquid). The model showed that the PSP CDS tablet had a significantly higher mean taste score than the liquid comparator (difference of 1.48 ± 0.66, *p*-value = 0.03) (Table 7). The oral liquid had a slightly higher variance in scores, but this was not significant. No significant variation between subjects was observed either. The order of administration of the two preparations was also not significant.

This taste study is the first to directly compare the taste masking effectiveness of a CDS formulation against a commercial oral liquid in a cross-over study where participants sampled both formulations in one sitting. The taste data indicate a higher effectiveness of the CDS formulation to mask the bitterness taste of PSP than the commercial liquid formulation. This was further confirmed when the participants were asked which formulation they would prefer to take again when unwell. A higher percentage of the participants (56%) chose the CDS tablet over the liquid formulation.

When participants were asked to provide a score for their willingness to take the formulation again, the mean scores were 7.32 ± 3.06 for the PSP CDS and 6.44 ± 2.86 for the oral liquid. However, there was no statistically significant difference between the groups (*p* = 0.176). The scores were converted to a binary preference variable based on the higher score, and a logistic regression model was fitted to these data. In this model, the odds of willingness to take the CDS tablet again were higher compared to taking the liquid formulation; however, the order of administration was significant. If the tablet was offered first then the odds for preferring the tablet over the liquid was lower (Odds ratio = 0.65, CI = (0.43, 0.97)).

**Figure 7:**
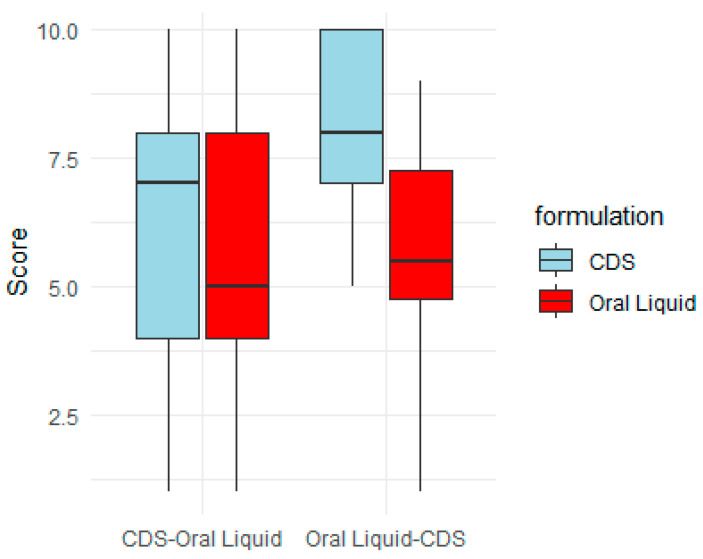
Order effect on taste score of prednisolone sodium phosphate (PSP) CDS tablet and PSP oral liquid. Total of 25 participants took part in taste evaluation. They were randomised into (1) CDS–oral liquid: participants received CDS tablet first, then oral liquid after 10 min washout (*n* = 13), and (2) oral liquid–CDS: participants received oral liquid first, then CDS tablet after 10 min washout (*n* = 12).

The box plot (Figure 7) further illustrates the effect of the order of taking the PSP CDS tablet and oral PSP liquid formulation in the taste evaluation. When the CDS tablet was randomised to be taken first, the median taste score was lower compared to when the tablet was randomised to be taken after the oral liquid (7 vs. 8). The interquartile range was also wider when the tablet was taken first, suggesting more variability in taste scores. The reason that the order of administration affected the taste score and preference for the PSP CDS tablet is not clear. Many participants had commented that the CDS and oral liquid formulations had aversive aftertastes, which agrees with a recent human taste evaluation study that found the bitterness taste of prednisolone aqueous solutions to linger even after the participants had gargled with water and waited for 5 min [25]. The aversive aftertastes might have accounted for the PSP CDS tablet, when it was taken first, to have a comparable taste profile to the oral liquid (Figure 7). Conversely, the improved taste score for the CDS tablet when it was taken second, after the liquid formulation, might have reflected a more favourable taste comparison with the liquid formulation. Unlike the CDS tablet, the taste score for the oral PSP liquid comparator was independent of the order of administration (median score of 5.5 when taken first vs. 5 when taken after the CDS tablet).

Previous paediatric clinical trials have indicated that the tramadol CDS tablet and midazolam CDS tablet also had significantly higher taste scores over their oral liquid comparators [7,8] (Table 7). PSP, like tramadol hydrochloride and midazolam hydrochloride, has a strong bitterness taste, and the CDS tablets of the three drugs were developed using similar inert ingredients. However, their taste evaluations were designed differently. The taste evaluation of PSP CDS tablets recruited young healthy adult participants and employed a cross-over design where the participants evaluated the CDS tablet and a commercial oral liquid as comparator in one sitting, and the drug dose was equivalent in both formulations. The taste evaluation of the tramadol CDS tablet and midazolam CDS tablet recruited child participants who were prescribed tramadol or midazolam in a preoperative clinical setting. To ensure there was no disruption to clinical management, each child participant was randomised to take either the CDS tablet or the liquid comparator to fulfil the prescribed drug dose. This created two issues in that the drug doses tested were not uniformly applied between participants and between formulations, as the drug doses were prescribed based on the body weight of the child participant. There was also no direct comparison of the taste acceptability of the CDS tablet formulation against the comparator formulation. Moreover, the liquid comparators in the midazolam CDS and tramadol CDS taste trials were pharmacy-compounded products that had not been optimised for taste masking. The comparator for the PSP CDS taste evaluation was Redipred™, which contained raspberry flavour and sorbitol as taste modifiers, and would have been manufactured in a GMP-certified facility. The PSP CDS taste evaluation is therefore a more rigorous comparison of the taste-masking effectiveness of the CDS formulation against a standard of care comparator formulation.

Nonetheless, a major limitation identified in the present study is the inadequacy of the taste evaluation design for drugs with aversive aftertastes. Published taste evaluation studies had reported utilising intervals that varied from 5 min to 20 min between the completion of evaluation of one sample and the commencement of evaluation of a second sample, while some studies do not specify a time interval [25,26,27]. The PSP CDS taste evaluation design was a randomised, controlled trial, and participants were instructed to cleanse their palate with water and plain crackers and to wait 10 min before taking the second formulation. Despite these precautions, many participants were unable to completely remove the residual aversive taste of PSP between formulations, suggesting that a longer wait period may be necessary to minimise crossover taste effects. An alternative is to employ a non-cross-over design where a participant would only evaluate the taste of one formulation and be provided with reference samples that do not have lingering aftertastes to guide baseline bitterness scoring. It may also be prudent to take multiple taste scores over a set period to evaluate medicinal formulations for their potential to mask not only the acute bitter tastes of drugs, but also residual aversive tastes. Unlike repeated measures studies, as was performed for the PSP CDS tablet in this study, these alternative approaches require a longer evaluation time frame and larger participant numbers to maintain statistical power and robustness of the study design. Identifying a more effective palate cleanser during the washout period could also be a valuable solution.

## 4. Conclusions

In summary, the CDS technology was transferable to preparing PSP CDS tablets that were chemically stable for at least 3 months at ambient conditions. Optimised tablets prepared at 50 °C and a stirring time of 26 h showed immediate drug release profiles in vitro. Compared to a commercial oral liquid PSP formulation, the optimised PSP CDS tablet provided more effective taste masking alongside convenience and accuracy of dosing, and better storage stability at ambient temperature. This study also successfully developed and validated a stability-indicating HPLC assay for the CDS formulations of PSP and PB. It further found PB to be unstable in the CDS formulation, and therefore not suitable for use in preparing prednisolone CDS tablets.

## 5. Patents

The CDS platform is patented under WO/2018/090096.

## Figures and Tables

**Table 1:** Formulations for prednisolone base CDS tablets and prednisolone sodium phosphate CDS tablets, each equivalent to 10 mg of prednisolone per tablet, and formulation for the control placebo CDS tablet.

Ingredients Name	Prednisolone Base (PB) CDS Tablet (mg/Tablet)	Prednisolone Sodium Phosphate (PSP) CDS Tablet (mg/Tablet)	Plain Placebo for Prednisolone CDS
Active drug	10	13.44	0
Sodium chloride	1.07	1.07	1.07
Steviol	2.14	2.14	2.14
Xanthian gum	5.34	5.35	5.34
Cremophor RH40	53.41	53.49	53.41
Polyethylene glycol 1450	106.82	106.98	106.82
Jamaica dark chocolate base	371.52	369.20	361.52
Total weight	550.30	551.67	530.30

**Table 2:** Gradient elution protocol for HPLC assay of prednisolone sodium phosphate and prednisolone base.

Time (min)	Solvent A (Acetonitrile) (%)	Solvent B (Buffer Containing 25 mM KH_2_PO_4_ and 0.25% Triethanolamine, pH 7.4) (%)
0–6	22	78
6–10	30	70
10–15	30	70
15–20	80	20
20–25	80	20
25–27	22	78
27–35	22	78

**Table 3:** Independent variables of the 2^3^ factorial design for the manufacture of prednisolone sodium phosphate CDS tablets.

Factors	−1.41	−1	0	1	1.41
Temperature (°C)	48	50	55	60	62
Stirring Time (h)	2	6	16	26	30

**Table 4:** Accuracy (recovery %) and precision (RSD %) of the HPLC assays for prednisolone sodium phosphate (PSP) and prednisolone base (PB).

% Concentration within Calibration Range	PSP		PB	
	Recovery (%)	RSD (%)	Recovery (%)	RSD (%)
50	99.50	1.44	99.74	0.61
75	98.17	0.10	99.36	0.75
100	99.41	1.35	99.48	1.47

**Table 5:** Residual drug content in 13.44 mg prednisolone sodium phosphate (PSP) CDS tablets and 10 mg prednisolone base (PB) CDS tablets upon storage for specified time points at ambient temperature and 40 °C. Drug content is expressed as a percent of baseline drug levels determined immediately after tablet manufacture. The baseline drug levels in the PSP CDS and PB CDS tablets were determined by the HPLC assay to be 99.4 ± 1.9% and 99.3 ± 0.3%, respectively, of labelled drug content. Data represents mean ± SD (*n* = 3).

Tablet Formulation	Storage Condition	Baseline	1 Month	3 Months
PSP CDS (% baseline content, mean ± SD, *n* = 3)	Ambient	99.4 ± 1.9	100.9 ± 2.8	98.6 ± 1.4
	40 °C	99.4 ± 1.10	99.5 ± 0.7	93.8 ± 2.1
PB CDS (% baseline content, mean ± SD, *n* = 3)	Ambient	99.3 ± 0.3	101.2 ± 1	89.4 ± 1.0
	40 °C	99.3 ± 0.4	77.1 ± 1	12.5 ± 2.9

**Table 6:** Design points and corresponding responses for tablet setting time.

Run	Temperature	Stirring Time	Response: Setting Time (in s)
1	1	−1	326
2	−1	1	116
3	0	0	315
4	−1	−1	114
5	1	1	344
6	0	0	315
7	0	0	315
8	1.41	0	392
9	0	−1.41	194
10	0	0	316
11	0	1.41	318
12	0	0	316
13	−1.41	0	112
14	0	0	316

**Table 7:** Taste evaluation of various formulations. Formulations include midazolam (MDZ), tramadol (TRM), and prednisolone sodium phosphate (PSP). CDS refers to the chocolate-based delivery system, and LQD refers to the oral liquid formulation. Taste evaluation was conducted using a 5-point hedonic scale for MDZ and TRM formulations and an 11-point scale for PSP formulations.

Formulation	MDZ CDS [7]	MDZ LQD [7]	TRM CDS [8]	TRM LQD [8]	PSP CDS	PSP LQD
Taste evaluation scale	5-point hedonic scale				11-point scale	
Taste Score (mean (SD))	3.19 (1.43)	1.71 (1.13)	3.69 (1.38)	2.00 (1.19)	7.08 (2.40)	5.60 (2.33)
Number of participants (*n*)	74	70	68	71	25	25

## Data Availability

Data are contained within the article.

## Appendix A. For Prednisolone Sodium Phosphate CDS Tablet

### Design of Experiments Data Analysis

A non-linear statistical model was found to be more appropriate for the data collected in the Design of Experiments (DoE) study. Consequently, we fitted a logistic model to the setting time against the independent variables of temperature and stirring time. The stirring time had insignificant effects on the setting time, and plots of the data showed a lack of any relationship. Three feasible models were fitted to the setting time with respect to temperature: a logistic model, an exponential model, and an advanced exponential model. All three seemed to fit well. The estimated model equations are as follows:

Logistic model:

$$Setting\;Time=\frac{368.8}{1+\exp\left(\frac{51.4-Temperature}{2.5}\right)}$$

Exponential model:

$$Setting\;Time=0.05\exp\left(0.145\;Temperature\right)+50$$

Advanced Exponential model:

$$Setting\;Time=2\times 2^{0.55\left(Temperature-48\right)}+110$$

All three models are plotted in Figure A1. The logistic model assumed that the setting time flattened off as temperature increased, while the exponential models assumed that the setting time continued to increase as temperature increased. Given that we are only interested in the model for the range of temperatures in the design space, we believe that the exponential model fits the data best. Figure A2 presents the diagnostic plots for the exponential model, demonstrating no biases.
